# Aldh2 and the tumor suppressor Trp53 play important roles in alcohol-induced squamous field cancerization

**DOI:** 10.1007/s00535-024-02210-y

**Published:** 2025-02-06

**Authors:** Yuki Kondo, Shinya Ohashi, Chikatoshi Katada, Yukie Nakai, Yoshihiro Yamamoto, Masashi Tamaoki, Osamu Kikuchi, Atsushi Yamada, Kenshiro Hirohashi, Yosuke Mitani, Shigeki Kataoka, Tomoki Saito, Trang H. Nguyen Vu, Tomohiro Kondo, Yu Uneno, Tomohiko Sunami, Akira Yokoyama, Junichi Matsubara, Tomonari Matsuda, Seiji Naganuma, Kohei Oryu, Samuel Flashner, Masataka Shimonosono, Hiroshi Nakagawa, Manabu Muto

**Affiliations:** 1 Department of Medical Oncology, Kyoto University Graduate School of Medicine, 54 Kawahara-Cho, Shogoin, Sakyo-Ku, Kyoto, 606-8507 Japan; 2 Department of Gastroenterology, Kitasato University School of Medicine, Sagamihara, Japan; 3 Preemptive Medicine and Lifestyle Disease Research Center, Kyoto University Hospital, Kyoto, Japan; 4 Environment Health Division, Kyoto University Graduate School of Engineering, Kyoto, Japan; 5 Faculty of Health Sciences, Department of Medical Laboratory Science, Kochi Gakuen University, Kochi, Japan; 6 Faculty of Health Sciences, Department of Nutrition, Kochi Gakuen University, Kochi, Japan; 7 Division of Digestive and Liver Diseases, Department of Medicine and Herbert Irving Comprehensive Cancer Center, Columbia University Irving Medical Center, New York, USA

**Keywords:** Field cancerization, SCC, Alcohol-drinking, Aldh2, TP53

## Abstract

**Background:**

Field cancerization defined by multiple development of squamous cell carcinomas (SCCs) in upper aerodigestive tract was explained by excessive alcohol intake. A dysfunctional mitochondrial aldehyde dehydrogenase 2 (Aldh2) delays the clearance of acetaldehyde, a genotoxic alcohol metabolite, and increases SCC risks. TP53 plays key roles in squamous carcinogenesis. However, the mechanism of alcohol-mediated squamous field cancerization has not been clearly elucidated.

**Methods:**

We developed a novel genetically engineered mouse strain *KTPA*^–/–^ (*Krt5Cre*^*ERT2*^*; Trp53*^*loxp/loxp*^*; Aldh2*^–/–^) featuring *Aldh2*-loss concurrent with epithelial-specific *Trp53* deletion. These mice were given 10%-EtOH, and we evaluated the development of squamous cell carcinogenesis histologically and genetically.

**Results:**

Widespread multifocal rete ridges (RRs), characterized by downward growth of proliferative preneoplastic cells, were found only in *Aldh2*^+*/*–^ and *Aldh2*^–*/*–^ mice with keratin5-specific Trp53 deletion (*KTPA*^+*/–*^ and *KTPA*^*–/–*^ mice, respectively), and alcohol drinking apparently increased RR formation rate. SCC occurred only in *KTPA*^*–/–*^ (*Aldh2* loss*/TP53* loss) mice with alcohol drinking (15/18: 83%). Total alcohol consumption volume was significantly higher in *KTPA*^*–/–*^ (*Aldh2* loss*/TP53* loss) mice with SCCs than those without SCCs. Further, target sequence revealed the occurrence of genetic abnormalities including *Trp53* mutations in the esophageal epithelium of *Aldh2*^*–/–*^ mice with alcohol drinking, suggesting direct mutagenic effects of alcohol drinking to the esophageal epithelium.

**Conclusion:**

This study provides for the first time the evidence that alcohol drinking, *Aldh2* dysfunction and *Trp53* loss cooperate in squamous field cancerization. Alcohol consumption volume affects the SCCs development, even in the same genotype.

**Supplementary Information:**

The online version contains supplementary material available at 10.1007/s00535-024-02210-y.

## Introduction

Multiple development of squamous cell carcinoma (SCC) in the upper aerodigestive tract has been explained by “Field cancerization” [1,2], and epidemiologic data suggest that alcohol-drinking is deeply involved in its pathophysiology [3,4]. However, direct evidence to support this phenomenon has not been established.

The International Agency for Research on Cancer has defined alcohol and acetaldehyde associated with alcohol consumption as definite human carcinogens [5,6]. Acetaldehyde is the first metabolite of ethanol (EtOH), and is detoxified by mitochondrial aldehyde dehydrogenase2 (Aldh2) [7,8]. In humans, > 8% of the global population and 30%–40% of East Asians carry a single nucleotide polymorphism *rs671*, Aldh2*2 that decreases the Aldh2 catalytic activity by > 100-fold compared with wild-type Aldh2*1 [8]. Aldh2*2 raises the risk for ESCC and other alcohol-related cancers in those who drink alcohol [9–11]. Aldh2 deficiency causes high acetaldehyde levels in the blood, saliva, and expired air following alcohol drinking [12–14]. Acetaldehyde causes genotoxic stress via mitochondrial dysfunction, to promote mutagenesis [15].

Mutations of the *TP53* tumor suppressor gene (*Trp53* in mice) are the most frequent genetic alterations in ESCC [16–18] and are found even in noncancerous esophageal epithelia of individuals with high alcohol consumption [19]. *TP53* mutations are also increased with age [19] and prevalent in preneoplastic mucosal lesions such as intraepithelial neoplasia [4,20].

Thus, alcohol consumption, inactivation of Aldh2 and *TP53* are epidemiologically proven to be deeply involved in the development of squamous carcinogenesis, however, it has not been elucidated in animal models whether these factors cooperate to contribute to squamous carcinogenesis and field effect. The aim of this study is to establish evidence that alcohol drinking causes multiple SCCs in the esophageal regions, and to clarify the pathophysiology of alcohol-induced squamous field carcinogenesis in those regions.

## Materials and methods

### Breeding and validation of experimental and control strains

All experiments conformed to the relevant regulatory standards and were approved by the Institutional Animal Care and Use Committee of Kyoto University (Med Kyo 21,312), and all sections of this report adhere to the ARRIVE Guidelines for reporting animal research.

To investigate the functional consequences of concurrent loss of *Aldh2* and *TP53* in the stratified squamous epithelia of the esophagus and adjacent upper aerodigestive mucosa, we crossed extensively characterized *Aldh2*-deficient (*Aldh2*^*–/–*^) mice [21,22] and *KTP* (*Krt5Cre*^*ERT2*^*; Rosa26tdTomato*^*loxP−stop−loxP/loxP−stop−loxP*^*; Trp53*^*loxP/loxP*^)mice [23], the latter featuring the cytokeratin Krt5 promoter-driven tamoxifen (TAM)-inducible Cre recombinase for squamous epithelial basal cell-specific *Trp53* deletion along with a cell-lineage tracing capability. (Supplementary Fig. 1A).

### Alcohol administration to mice

Age-matched (6–8-week-old) male mice were allowed to drink ad libitum water supplemented with or without 10% vol. EtOH. Alcohol exposure was initiated 2 weeks after treatment of mice with or without TAM. Mouse weight was monitored once a week, and the water feeding bottles were changed once a week. The amount (g) of alcohol consumption was calculated as follows: the volume of water consumed in one week (mL) × alcohol content in 10% vol EtOH (0.1) × specific gravity (0.8) (g/mL). Alcohol was discontinued when mice lost weight by 20% according to the regulations of the animal facility. Mice were then euthanized and dissected for organ collection. As for the mouse experiments, the following two cohorts were tested. In Cohort 1 (*n* = 104), alcohol was administered continuously without a break. In Cohort 2 (*n* = 18), TAM-treated *KTPA*^*–/–*^ (*TP53*^*–/–*^; *Aldh2*^*–/–*^) mice were treated with alcohol. When 20% or more body weight loss occurred due to the continuous alcohol drinking, drinking fluid was replaced with water, and then the mice were allowed to drink alcohol again after body weight regained.

### Histopathological and immunohistochemical assessment

Mice were euthanized painlessly under anesthesia with diethyl ether inhalation followed by cervical dislocation. For autopsy, the esophagus and stomach were removed, incised in the longitudinal direction. Tissue samples were fixed in 10% neutral buffered formalin (FUJIFILM) overnight, embedded in paraffin, and cut into 4-µm sections for standard H&E staining and immunohistochemistry. Histopathological evaluation regarding SCC and/or IEN (LGIEN/HGIEN) occurrence was diagnosed by a pathologist (SN) and defined as follows [24,25]: LGIEN, mild cytologic atypia, and limits to the lower half of the squamous epithelium of the esophagus; HGIEN, more than half the squamous epithelium involved by cytologic atypia or in the presence of severe cytologic dysplasia; SCC, squamous malignancy with invasion of basement membrane. Depending on the cytologic features, cytologic atypia, mitotic activity, and extent of keratinization were included.

### Rete ridge counts

Formation of Rete ridges (RRs), a drop-shaped downward expansion of the proliferative basal cell compartment within the squamous epithelia [26–30], were evaluated histologically using epithelia (2000 µm) of the esophagus and forestomach of 12 groups of mice (*n* = 104) divided by *Trp53*, *Aldh2* deficiency, and alcohol-drinking status, respectively. RRs were counted as a single RR if they had a drop-shaped morphology and an epithelium that was more than twice as thick as the smallest epithelium in the range. The presence of cellular atypia or polarity disorder was not considered when counting RRs.

### Colony formation assay

Immortalized normal human esophageal keratinocytes, EPC3-hTERT, were cultured in fully supplemented Keratinocyte Serum-free media (KSFM, Thermo Fisher Scientific) as described [31]. Cells were treated with acetaldehyde (0 or 0.2 mM) (Sigma-Aldrich) for 24 weeks. The cell-Matrigel suspensions were cultured in fully supplemented KSFM supplemented with 10-µM SB431542 (TOCRIS1614, bio-techne) and 10-µM Y-27632 (TOCRIS1254, bio-techne) for 7 days [32]. The number and size of proliferating colonies in the high-power field were counted using a microscope (ECLIPSE Ti-S, Nikon), as previously shown [33].

### Target gene sequence of the esophageal epithelium in *Aldh2* knockout mice with alcohol drinking

Five-week-old male *Aldh2*^–/–^ mice were obtained from the Department of Environmental Health, University of Occupational and Environmental Health (Kitakyushu) [21]. After 12 weeks of drinking 10% EtOH in water or water alone, they were sacrificed, and then esophageal tissues were harvested by peeling away from the submucosal tissues using tapered tweezers under a microscope (BX53, OLYMPUS). To obtain mouse genomic DNA, esophageal tissues were lysed using Cell Lysis Solution (10-mM Tris–HCl pH8.0, 5-mM EDTA, 0.5%(W/V) SDS) with Proteinase K. To remove impurities, the lysate was treated with RNase A at a final concentration of 1.3 mg/mL, followed by protein precipitation by adding EDTA and ammonium acetate at final concentration of 0.25 mM and 2 M, respectively. Highly purified DNA was obtained by isopropanol precipitation, followed by additional purification using DNA Clean & Concentrator Kit (Zymo Research). DNA concentrations were measured using Qubit dsDNA BR Assay Kits (Invitrogen). Then, target capture sequencing was performed as follows. Capture probes targeting coding exons and untranslated regions of six mouse genes (*Trp53*, *Notch1*, *Notch3*, *Pik3ca*, *Cdkn2a,* and *Fat1*), which were orthologs of major hotspot genes in human ESCC [34], were designed using SureDesign (Agilent technologies). Library samples were prepared using Agilent SureSelect XT HS reagents (Agilent technologies) by following the manufacturer’s protocol. A total amount of 200 ng of genomic DNA was processed by Covaris M220 shearing system (Covaris) to obtain DNA fragments of about 200 base pairs, followed by steps of end repairing, adapter ligation, and capture hybridization. In quality control and quantification of library samples, TapeStation2200 (Agilent technologies) was used. Ultra-deep sequencing was carried out by paired-end mode on MiniSeq system (Illumina) with high output kit (300 cycles). Sequencing data was analyzed using the following pipelines. Preprocessing of FASTQ files was carried out using Fastp (v0.23.2) [35] to remove adapter sequence and low-quality reads (quality score < 15, read length < 15). Read alignment to mouse reference genome (mm9) was performed using Burrows-Wheeler Aligner (BWA) MEM algorithm v0.7.17 [36]. Duplicate reads in aligned sequences were removed based on information of unique molecular identifiers using AGeNT LocatIt (v4.0.1, Agilent Technologies). Local read alignment and base quality scores were recalibrated using ABRA (v0.97) [37] and the Genome Analysis Toolkit (v4.1.0.0) [38], respectively. Then, short variants were called using MuTect2 (v4.1.0.0) [39] and were annotated using SnpEff (v4.3) [40]. SAMtools (v1.9) and BCFtools (v1.10.2) [41] were used to manipulate data of aligned sequences and variant call format, respectively. Depth of coverage were counted using bam-readcount (v0.8.0) and over 14,000 of average depth of deduplicated reads were confirmed for each sample. Sample-specific variants with > 0.1% allele fraction were included in the mutation analysis (Supplementary Table 1).

### Statistical analyses

Data are presented as the mean ± standard deviation (SD), unless otherwise stated. Data were analyzed by the 2-tailed Student t test between two groups or one-way ANOVA followed by Tukey’s multiple comparison among three or more groups, unless otherwise indicated. Correlations were assessed using Pearson’s correlation test. Statistical analyses were performed using GraphPad Prism (version 9). P-values of less than 0.05 were considered significant.

Materials and methods for supplement figure details are described in supplementary materials.

## Results

### Generation of KTPA strains with Trp53 conditional alleles and Aldh2 deficiency

Genetic characterization in each KTPA strain mice is shown in Table 1.a. First, *TP53* deficiency in the stratified epithelia of the esophagus and forestomach as well as Aldh2 activity in *KTPA* strains (*KTPA*^+*/*+^ [*TP53*^*–/–*^; *Aldh2*^+*/*+^), *KTPA*^+*/–*^ [*TP53*^*–/–*^; *Aldh2*^+*/–*^], and *KTPA*^*–/–*^ [*TP53*^*–/–*^; *Aldh2*^*–/–*^]) was investigated. As shown in Supplementary Fig. 1b and 1c, TAM administration in *KTPA* strains resulted in Cre-mediated *Trp53* deletion and concurrent induction of tdTomato fluorescent protein in the esophagus and the forestomach, but not the glandular (distal) stomach, thus we confirmed *Trp53* deletion in TAM-treated *KTPA* strains. As for Aldh2 activity, *KTPA*^*–/–*^ (*TP53*^*–/–*^; *Aldh2*^*–/–*^) mice showed minimal *Aldh2* activity whereas *KTPA*^+*/–*^ (*TP53*^*–/–*^; *Aldh2*^+*/–*^) mice showed an intermediate Aldh2 activity compared with *KTPA*^+*/*+^ (*TP53*^*–/–*^; *Aldh2*^+*/*+^) mice (Supplementary Fig. 1d), thus we confirmed functional *Aldh2* deficiency in the experimental strains *KTPA*^*–/–*^ (*TP53*^*–/–*^; *Aldh2*^*–/–*^) and *KTPA*^+*/–*^ (*TP53*^*–/–*^; *Aldh2*^+*/–*^). Genotyping of each mice strain regarding *Aldh2*, *Trp53*, *Krt5-Cre*, *R26td Tomato* gene was strictly confirmed, as shown in Supplementary Fig. 2.

**Table 1.** Characteristics of KTPA mice, experimental groups and tumor formation rate (a) Genetic characterization in each *KTPA* strain mice. (b) Treatment regimen and tumor incidence for each *KTPA* strain mice (Cohort1)

(a) Genetic character	*KTPA*^+/+^ mice	*KTPA*^+/^^–^ mice	*KTPA*^–^^/^^–^ mice
*Trp53*	(–/–)	(–/–)	(–/–)
*Aldh2*	(+/+)	(+/+)	(–/–)
(b) Tumor incidence	*KTPA*^+/+^ mice	*KTPA*^+/^^–^ mice	*KTPA*^–^^/^^–^ mice
TAM(–)：Trp53^+/+^Water	0/5	0/5	0/5
TAM(+)：Trp53^－/－^Water	0/10	0/10	0/10
TAM(–)：Trp53^+/+^10% EtOH	0/10	0/10	0/10
TAM(+)：Trp53^–^^/^^–^10% EtOH	0/9	0/10	3/10

### Induction of IEN and SCC in alcohol-fed *KTPA*.^*–/–*^ mice: Study Cohort 1

In study cohort 1, we utilized *KTPA*^+*/*+^ (*TP53*^*–/–*^; *Aldh2*^+*/*+^), *KTPA*^+*/–*^ (*TP53*^*–/–*^; *Aldh2*^+*/–*^), and *KTPA*^*–/–*^ (*TP53*^*–/–*^; *Aldh2*^*–/–*^) mice in four groups per strain (with or without TAM, and/or with or without 10% EtOH) (Table 1.b). Briefly, in two groups, mice were given TAM (200 mg/kgBw) via oral gavage to delete *Trp53*. The other two groups received vehicle control (corn oil). Two weeks later, two groups started ad libitum drinking water containing 10% EtOH. The other two groups were not exposed to EtOH. All mice, except those that displayed a sign of morbidity or a loss of weight by 20%, were euthanized at the end of the 50-week alcohol treatment period and dissected to collect organs for histopathological examination. The duration that TAM-treated *KTPA*^*–/–*^ (*TP53*^*–/–*^; *Aldh2*^*–/–*^) mice could continue to drink EtOH varied (22–50 weeks) among individuals based on their health conditions. *KTPA*^*–/–*^ (*TP53*^*–/–*^; *Aldh2*^*–/–*^) mice without TAM treatment were able to sustain EtOH drinking for 50 weeks. All alcohol-unfed *KTPA*^*–/–*^ (*TP53*^*–/–*^; *Aldh2*^*–/–*^) mice remained healthy with or without TAM treatment. All *KTPA*^+*/–*^ (*TP53*^*–/–*^; *Aldh2*^+*/–*^) and *KTPA*^+*/*+^ (*TP53*^*–/–*^; *Aldh2*^+*/*+^) mice remained healthy regardless of the treatment conditions (with or without EtOH drinking and/or TAM treatment).

**Fig. 1:**
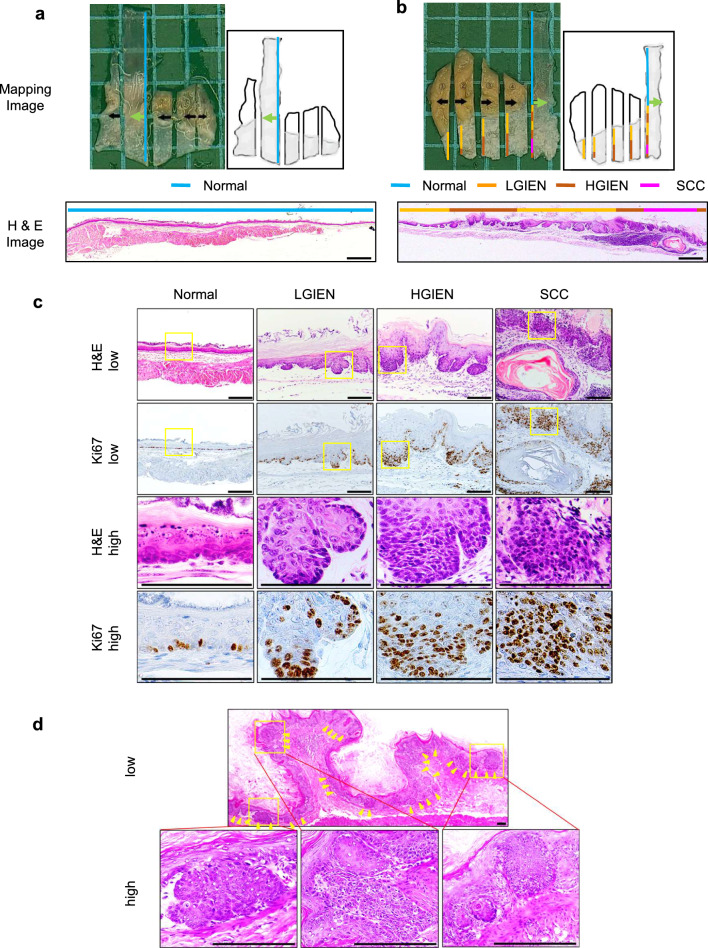
Alcohol drinking induces IEN and SCC in TAM-treated *KTPA*^–/–^ (*TP53*^*–/–*^; *Aldh2*^*–/–*^) mice. **a** and **b** Representative macroscopic and microscopic images of dissected esophagus and forestomach samples were collected from TAM-treated *KTPA*^–/–^ (*TP53*^*–/–*^; *Aldh2*^*–/–*^) mice that received drinking water containing no EtOH for 34 wk in a or 10% EtOH for 44 wk in (**b**). The specimens were grossed and serially sectioned in the direction indicated by black arrows. Each section was subjected to histopathological mapping of normal, low-grade IEN (LGIEN), high-grade IEN (HGIEN), and SCC lesions as indicated by a color code. Green arrows indicate directions of cross sections for the representative H&E-stained slides shown. **c** Representative H&E- and Ki67-stained slides of normal, LGIEN, HGIEN, and SCC lesions. The area demarcated by yellow rectangles was enlarged in the lower panels. **d** Multifocal IEN and invasive SCC lesions (yellow arrowheads). Invasive tumor fronts demarcated by yellow rectangles in the upper panel were enlarged in the lower panels. Scale bars = 0.5 mm in (**a** and **b**), and 100 µm in (**c** and **d**)

Regarding histology in the Cohort1 study (experiment of drinking alcohol continuously without a break period), no TAM-treated and alcohol-unfed *KTPA*^*–/–*^ (*TP53*^*–/–*^; *Aldh2*^*–/–*^) mice displayed neoplastic changes (Fig. 1a), however, three out of ten TAM-treated and alcohol-fed *KTPA*^*–/–*^ (*TP53*^*–/–*^; *Aldh2*^*–/–*^) mice (30%) harbored preneoplastic IEN or SCC within the squamous epithelium of the forestomach (Fig. 1b; Table 1.b). Mice in the other groups did not show neoplastic changes in the esophagus and forestomach, regardless of whether they drank alcohol or not, and/or they received TAM treatment or not (Table 1.b). In all groups of mice, there were no apparent neoplastic lesions in the distal (glandular) stomach, and other organs including the liver and the intestine (data not shown).

Both IEN and SCC lesions showed aberrant proliferation as evidenced by an increase in Ki67-positive basaloid cells (Fig. 1c). One of the unique features of this mouse model is that CreERT2-driven tdTomato expression under the *Krt5* promoter allows cell-lineage tracing following TAM administration. The neoplastic lesions displayed an increased tdTomato expression, and they were recognized as a pinky lesion (Supplementary Fig. 3b and c), in line with abnormal epithelial thickening of the esophagus and the forestomach linked to aberrant proliferation with Ki67 positive cells (Supplementary Fig. 3d, e, f, g and h). In addition, tdTomato expression indicated that alcohol-induced IEN and SCC cells originate from Krt5 + basal cells.

Examination of the entire esophagus and forestomach revealed the presence of multifocal squamous epithelial lesions compatible with low-grade IEN (LGIEN), high-grade IEN (HGIEN), or SCC with various degrees of atypia in the esophagus and forestomach (Fig. 1c). Notably, multiple and separate microinvasive SCC lesions were found within the forestomach (Fig. 1d). These findings suggest that concurrent *Trp53* loss and *Aldh2* dysfunction cooperate in alcohol-induced squamous field cancerization.

### Rete ridges as an early morphologic change in alcohol-induced SCC development

**Fig. 2:**
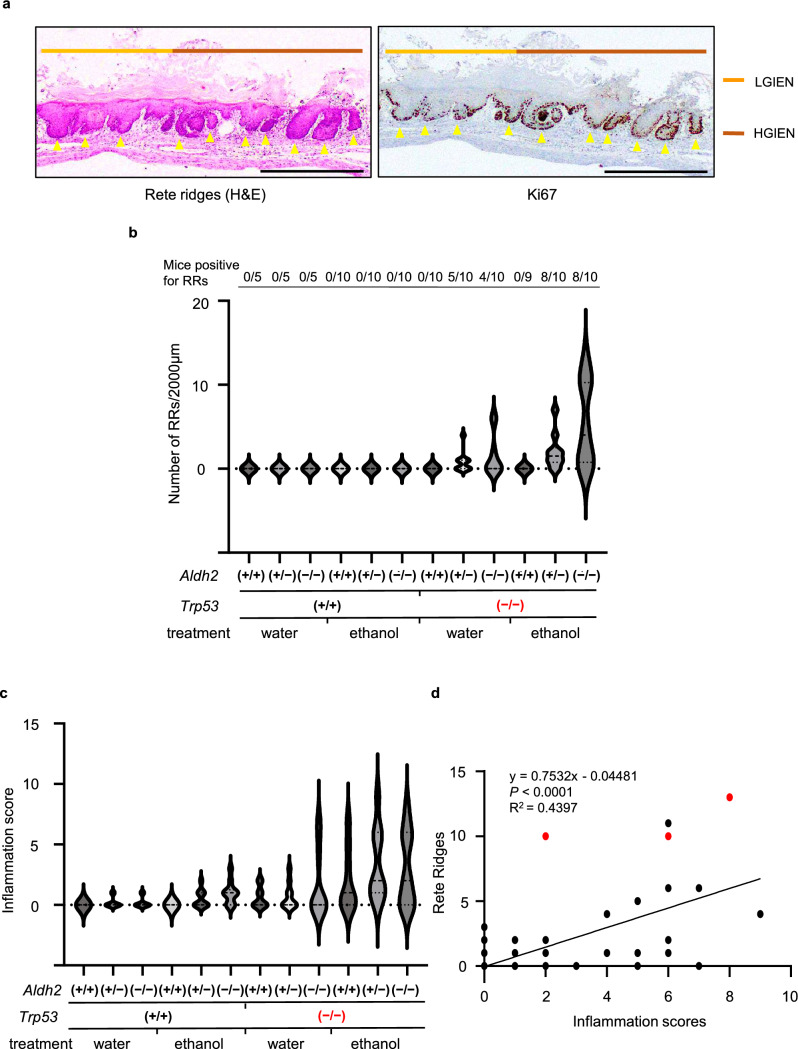
Rete ridges may be linked to mucosal inflammation during alcohol-induced carcinogenesis. **a** Representative H&E and immunohistochemistry images capturing rete ridges (RRs) (yellow arrowheads) characterized by highly proliferative Ki67 + cells and drop-shaped downward growth in the forestomach from alcohol-fed TAM-treated *KTPA*^–/–^ (*TP53*^*–/–*^; *Aldh2*^*–/–*^) mice. Scale bars = 1 mm. **b** The number of RRs per 2 mm of the squamous epithelial basal segment is shown in the violin plots for all mouse strains and indicated treatment conditions, with the frequency for each group indicated at the top. **c** Inflammation scores plotted for all mouse strains and indicated treatment conditions. **d** Scatter plot showing the relationship between the number of RRs and inflammation scores of all strains and conditions. Red circles indicate the mice that developed IEN /SCC, whereas black circles indicate the mice with normal epithelium. *n* = 104, *r* = 0.6631, *P* < 0.0001, red circles vs black circles. The dotted lines represent the 95% confidence intervals for the linear regression

In this study, we focused on the appearance of RRs, a drop-shaped downward expansion of the proliferative basal cell compartment within the squamous epithelia that are a histopathologic feature of atypical epithelia implicated in SCC development [26–30], as an initial morphologic change after long-term alcohol drinking. As shown in Fig. 2a, RRs were detected in IEN lesions with varying degrees of atypia and featured Ki67-positive basaloid cells indicative of proliferation (Fig. 2a).

Further, we assessed the RR formation rate in *KTPA*^+*/*+^(*TP53*^*–/–*^; *Aldh2*^+*/*+^), *KTPA*^+*/–*^ (*TP53*^*–/–*^; *Aldh2*^+*/–*^), and *KTPA*^*–/–*^ (*TP53*^*–/–*^; *Aldh2*^*–/–*^) mice treated with or without TAM and/or with or without EtOH drinking (Fig. 2b). Without TAM-induced *Trp53* deletion, RRs were absent regardless of *Aldh2* status or EtOH intake. Of note, TAM-treated *KTPA*^*–/–*^ (*TP53*^*–/–*^; *Aldh2*^*–/–*^) and *KTPA*^+*/–*^ (*TP53*^*–/–*^; *Aldh2*^+*/–*^), but not *KTPA*^+*/*+^( *TP53*^*–/–*^; *Aldh2*^+*/*+^), mice displayed an increased RR formation without EtOH drinking. Indeed, the RR formation rate was elevated without EtOH exposure in 5 out of 10 *KTPA*^+*/–*^ (*TP53*^*–/–*^; *Aldh2*^+*/–*^) and 4 out of 10 *KTPA*^*–/–*^ (*TP53*^*–/–*^; *Aldh2*^*–/–*^) mice following TAM treatment. Interestingly, EtOH drinking further increased RR formation in both *KTPA*^+*/–*^ (*TP53*^*–/–*^; *Aldh2*^+*/–*^) (8/10 mice) and *KTPA*^*–/–*^ (*TP53*^*–/–*^; *Aldh2*^*–/–*^) mice (8/10 mice) following TAM treatment. Importantly, the RR formation rate was highest (max 13, median 4) in TAM-treated and alcohol-fed *KTPA*^*–/–*^ (*TP53*^*–/–*^; *Aldh2*^*–/–*^) mice.

RRs may permit increased crosstalk between epithelial cells and subepithelial stromal components such as immune cells to foster the tumor-promoting microenvironment. Therefore, we assessed inflammatory cell infiltration in each treatment group of mice. As shown in Fig. 2c and Supplementary Fig. 4a, b, c and d, there was little inflammatory cell infiltration in the group of mice without TAM-induced *Trp53* deletion. Interestingly, TAM-treated mice displayed increased inflammation regardless of EtOH exposure or the *Aldh2* status, suggesting that Trp53 and Aldh2 may cooperate to suppress the inflammatory microenvironment. Moreover, there was a strong correlation (*r* = 0.7299, *P* < 0.0001) between RR formation and inflammation scores when all mice (*n* = 104) were included (Fig. 2d).

### SCC development in KTPA^–/–^ (*TP53*^*–/–*^; *Aldh2*^*–/–*^) mice depends upon the total amount of alcohol consumption

**Fig. 3:**
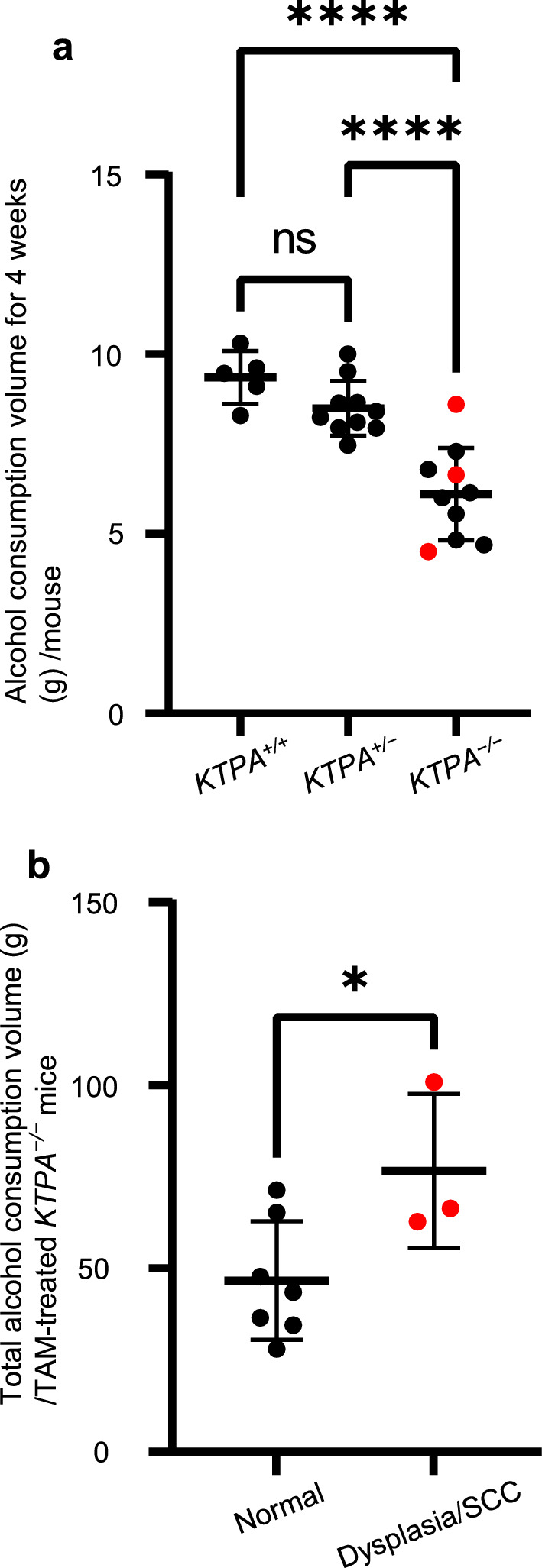
Alcohol consumption and esophageal carcinogenesis in *KTPA*^–/–^ (*TP53*^*–/–*^; *Aldh2*^*–/–*^) mice. **a** Alcohol consumption during the initial 4 wk after the start of alcohol drinking was plotted for TAM-treated *KTPA*^–/–^ (*TP53*^*–/–*^; *Aldh2*^*–/–*^) (*n* = 10), *KTPA*^+/–^ (*TP53*^*–/–*^; *Aldh2*^+*/–*^) (*n* = 10), and *KTPA*^+/+^ (*TP53*^*–/–*^; *Aldh2*^+*/*+^) (*n* = 5) mice with indicated Aldh2 status. One-way ANOVA (*P* < 0.05), followed by post hoc Tukey’s multiple comparison test. Error bars represent mean ± SD. ****P < 0.0001. **b** The total alcohol amount consumed by TAM-treated *KTPA*^–/–^ (*TP53*^*–/–*^; *Aldh2*^*–/–*^) (*n* = 10) was plotted for the entire alcohol-drinking period. 2-Tailed Student t test. Error bars represent mean ± SD. *P < 0.05. In (**a**–**b**), each circle represents a single mouse. Red circles indicate mice with IEN or SCC mice, and black circles indicate mice with normal mucosa mice

We assessed the correlation between alcohol consumption volume and the incidence of IEN or SCC in TAM-treated and alcohol-fed *KTPA* derivatives in Cohort1 study. The four weeks alcohol consumption volume in *KTPA*^*–/–*^ (*TP53*^*–/–*^; *Aldh2*^*–/–*^) mice was significantly lower (6.1 ± 1.3 g) than *KTPA*^+*/*+^ (*TP53*^*–/–*^; *Aldh2*^+*/*+^) and/or *KTPA*^+*/–*^ (*TP53*^*–/–*^; *Aldh2*^+*/–*^) mice (9.4 ± 0.7 g and 8.5 ± 0.8 g, respectively) (Fig. 3a), indicating that *KTPA*^*–/–*^ (*TP53*^*–/–*^; *Aldh2*^*–/–*^) mice have a decreased EtOH tolerance compared with *KTPA*^+*/–*^ (*TP53*^*–/–*^; *Aldh2*^+*/–*^) and *KTPA*^+*/*+^ (*TP53*^*–/–*^; *Aldh2*^+*/*+^) mice. Heterozygous *Aldh2* loss did not affect alcohol consumption in *KTPA*^+*/–*^ (*TP53*^*–/–*^; *Aldh2*^+*/–*^) mice where no neoplastic lesions developed, suggesting a potential species difference between mice and humans.

We next assessed the relationship between the incidence of IEN or SCC and alcohol consumption volume among TAM-treated *KTPA*^*–/–*^ (*TP53*^*–/–*^; *Aldh2*^*–/–*^) mice. Total alcohol consumption volume was significantly higher in mice with IEN or SCC than those without (Fig. 3b) (*P* = 0.0386).

### Long-term effects of alcohol drinking in TAM-treated *KTPA*^*–/–*^ (*TP53*^*–/–*^; *Aldh2*^*–/–*^) mice: Study Cohort 2

To examine the long-term effects alcohol drinking in mice, we performed the Cohort 2 experiments using TAM-treated *KTPA*^*–/–*^ (*TP53*^*–/–*^; *Aldh2*^*–/–*^) mice. Here, mice that lost 20% of their body weight loss were given breaking period (water drinking), and then allowed to drink EtOH again after regaining body weight. This method made it possible to drink EtOH for a long period of time (median: > 34 weeks) in TAM-treated *KTPA*^*–/–*^ (*TP53*^*–/–*^; *Aldh2*^*–/–*^) mice.

**Fig. 4:**
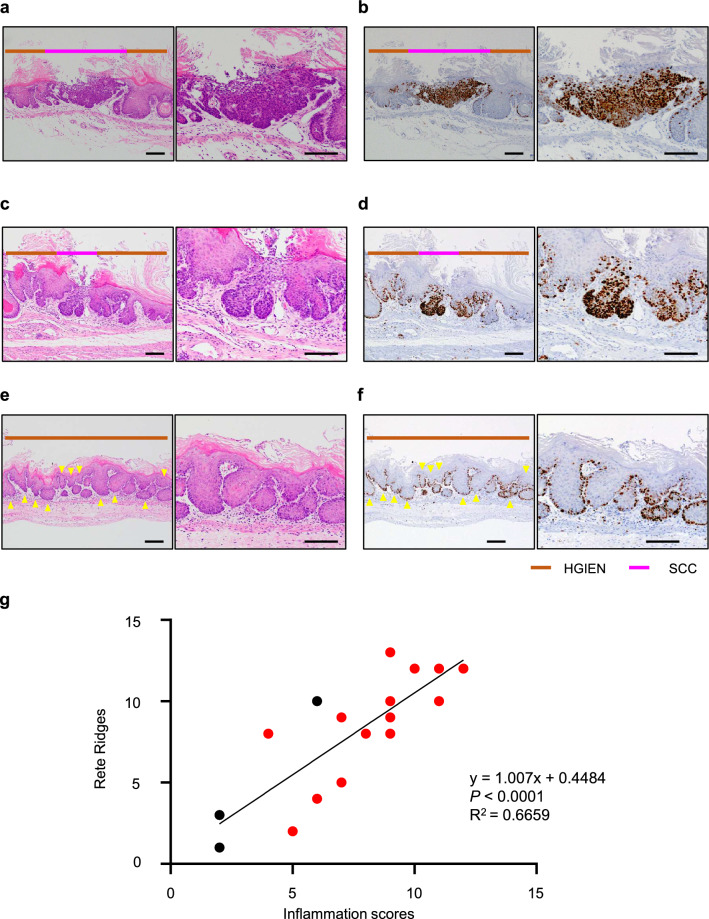
TAM-treated and alcohol-fed *KTPA*^–/–^ (*TP53*^*–/–*^; *Aldh2*^*–/–*^) mice induce SCC development at high rates by Cohort 2 study. **a**, **b** Representative H&E- and Ki67-stained images with SCC. The right panel were enlarged in the left panels. **c**, **d** Representative H&E- and Ki67-stained images of tumor area with strong inflammatory changes. The right panel were enlarged in the left panels. **e**, **f** Representative H&E- and Ki67-stained images of rete ridges (yellow arrowheads) with strong inflammatory changes. Scale bars = 100 µm (**a, b, c, d, e** and **f**). **g** Scatter plot showing the relationship between the number of RRs and inflammation scores of alcohol-fed TAM-treated *KTPA*^–/–^ (*TP53*^*–/–*^; *Aldh2*^*–/–*^) mice. Red circles indicate the mice developed IEN/SCC, whereas black circles indicate the mice with normal epithelium. *n* = 18, *r* = 0.8160, *P* < 0.0001, red circles vs black circles

As a result, 15/18 (83%) mice showed dysplasia/ESCC changes (Fig. 4a and b). Similar to Cohort 1, RR formation and inflammatory cell infiltration were also observed in mice that had been fed EtOH (Fig. 4c, d, e and f), and there was a strong correlation (*r* = 0.8160, *P* < 0.0001) between RR formation and inflammatory cell infiltration (Fig. 4g).

### Long-term effects of acetaldehyde exposure upon epithelial cell colony formation

**Fig. 5:**
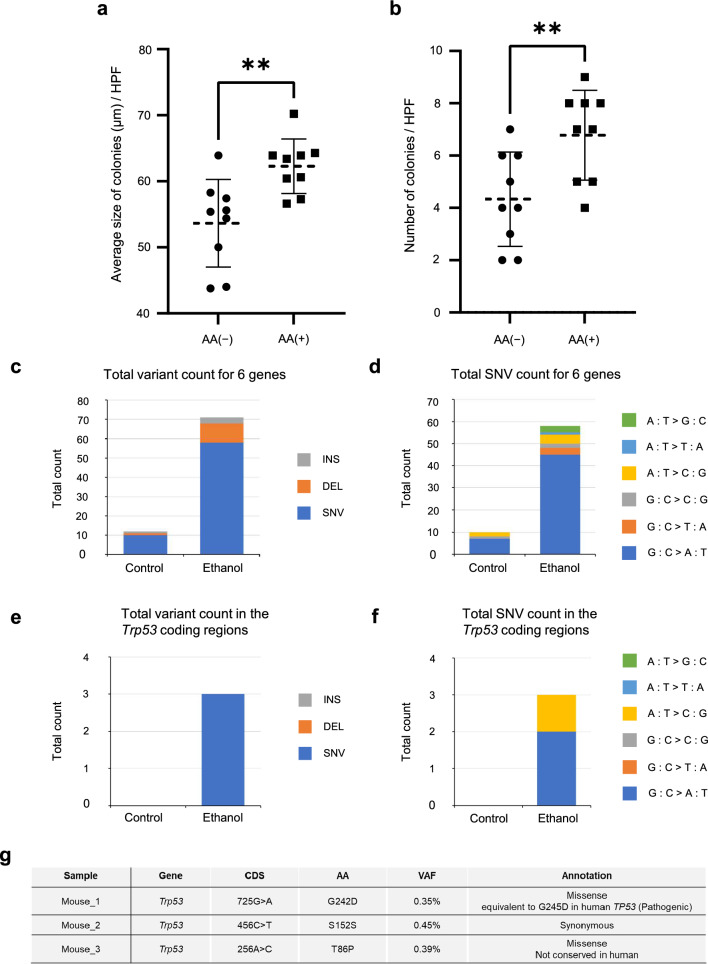
Effects of long-term acetaldehyde exposure on esophageal epithelial cells in vitro and effects of long-term alcohol drinking on *Aldh2*-deficient mice in vivo. EPC3-hTERT cells were cultivated for 24 weeks in a medium supplemented with or without 0.2 mM acetaldehyde (AA), and then those cells were subjected to colony formation assays. The average size (**a**) and number (**b**) of colonies were determined under the high-power field. *n* = 9, Error bars represent mean ± SD. **P < 0.01. Target sequencing of esophageal epithelium in *Aldh2*^*ko/ko*^ mice treated with 10% EtOH or water (Control) for 3 months was conducted (Control, n = 3; EtOH, *n* = 3). **C** Total count of short variants (variant type-specific: single nucleotide variant (SNV), insertion (INS), deletion (DEL)) in 3 mice for 6 genes (*Trp53, Notch1, Notch3, Pik3ca, Cdkn2a, and Fat1*). **d** Total count of SNVs (base substitution pattern-specific) in 3 mice for 6 genes. **e** Total count of short variants in 3 mice for *Trp53* coding regions. **f** Total count of SNVs in 3 mice for *Trp53* coding regions. **g** Detection of three SNVs in the *Trp53* coding regions in *Aldh2*-deficient mice with alcohol drinking for 3 months

Next, we evaluated whether acetaldehyde treatment may directly alter the biologic properties of esophageal epithelial cells in vitro. As shown in Figure 5a and b, long-term (24 weeks) acetaldehyde exposure significantly increased the size and number of esophageal epithelial colonies, suggesting that acetaldehyde increase the proliferative capability of squamous epithelial cells.

### Effects of alcohol drinking on genetic abnormalities in the esophagus of mice with Aldh2 deficiency

Lastly, we investigated whether alcohol drinking induces genetic mutations in the esophagus of mice with *Aldh2* deficiency. Here, *Aldh2* knockout mice were allowed to drink 10% EtOH or water for 3 months. We assessed whether alcohol exposure promotes the mutation of ESCC-associated genes. We selected 6 mouse gene orthologues (*Trp53*, *Notch1*, *Notch3*, *Pik3ca*, *Cdkn2a,* and *Fat1*), whose orthlogues are recurrently mutated in human ESCC [34], as targets for high resolution targeted deep sequencing. Target sequence data demonstrated that EtOH drinking increased the total count of short variants in the esophagus of *Aldh2*-deficient mice with alcohol drinking (Fig. 5c). As for single nucleotide variants (SNVs) in target sequence data, greater number of SNVs were detected in *Aldh2*-deficient mice with alcohol drinking than in those without alcohol drinking (Fig. 5c). Among SNVs, G to A (C to T) substitutions were recurrently observed (Fig. 5d). When focusing on the *Trp53* gene, greater number of short variants were also detected in *Aldh2*-deficient mice with alcohol drinking than in those without alcohol drinking (Fig. 5e), and substitution pattern were shown in Fig. 5f. Of note, we detected three *Trp53* SNVs　(Fig. 5f), one of which was a missense variant G242D (c.725G > A) and was presumed to be oncogenic on the basis of its homology to the hotspot variant G245D in human *TP53* [42,43].

## Discussion

Squamous field cancerization in the upper aerodigestive tract has been clinically well recognized, however, its mechanism has not been clearly understanded. In this study, we demonstrate for the first time that the combination of alcohol drinking *Aldh2* deficiency and *Trp53* deletion contributes to squamous field cancerization in animal models. Although there have been other animal ESCC models using other carcinogens (4NQO) in the past [23,44], our study is the first report to demonstrate that alcohol consumption causes ESCC in animals.

In this study, neoplastic changes occurred exclusively in the forestomach, but not in other regions in *KTPA*^*–/–*^ (*TP53*^*–/–*^; *Aldh2*^*–/–*^) mice, although human ESCC occurs throughout the entire esophagus, from the cervical esophagus to the abdomen. We speculate that the lack of neoplastic lesions in these regions may be due to the differential anatomic structures between mice and humans. As mice are four-legged animals, alcohol may be more likely to accumulate in the forestomach, and alcoholic liquid retention during alcohol drinking may differ between mice and humans. Therefore, we suggest that the difference in the exposure duration of alcohol and/or acetaldehyde to the mice esophagus and forestomach may have influenced the rate of tumor formation. The mouse forestomach is derived from squamous epithelium and forestomach is considered to be an extension of the esophagus [45,46], so that we believe this study contribute to elucidate the mechanism of alcohol-induced esophageal carcinogenesis.

In this study, widespread RR formation was shown in the squamous epithelium of the mice with *Aldh2* deficiency and *Trp53* deletion, while RR formation was not shown in *Aldh2* wild-type mice. Furthermore, IEN and SCC were induced only in alcohol-fed *Aldh2* ± *and -/-* mice with *Trp53* loss. None of the mice developed IEN or SCC without having alcohol drinking together with concurrent *Aldh2* dysfunction and *Trp53* deletion, suggesting that they are all essential for SCC pathogenesis through delayed acetaldehyde clearance and genome instability. We believe that this result faithfully reproduces the development of ESCC and field cancerization.

Clinically, RRs are frequently identified in the background squamous epithelium of the patients with ESCC as feature of structural abnormalities in atypical lesions of squamous epithelium [28]. Our result experimentally demonstrated that RRs were the early atypical changes, mimicking the pathology of squamous atypia, in squamous epithelium associated with alcohol drinking. In terms of pathophysiology, RRs are associated with mechanical strength and homeostasis of stratified squamous epithelia [47], and RRs are a feature of epidermal regeneration in response to repetitive mechanical insults [48,49]. The depth and architecture of RRs reflect the quality of wound repairs [50,51]. Therefore, we speculate RR formation may reflect chronic esophageal epithelial injury as well as active regeneration during esophageal carcinogenesis. We believe that widespread RRs changes are the key to develop field cancerization, because *Trp53* loss and *Aldh2* dysfunction might cooperate in RRs formation in preneoplastic lesions. In addition, we have also observed a strong correlation between RR formation and inflammatory cell infiltration, which is consistent with previous work linking esophageal carcinogenesis to the inflammatory tumor microenvironment [52]. Although our study suggests that TP53-mediated cell-cycle checkpoint function and Aldh2-mediated aldehyde clearance limit the aberrant proliferation of basal cell populations in the squamous epithelia, further investigation is warranted to elucidate the role of RRs in alcohol-induced field carcinogenesis.

The important implication of our data is that total alcohol consumption volume influences SCC development even amongst individuals with a similar genetic background. Our data corroborate the notion that heavy alcohol drinking increases the risk of ESCC in individuals with dysfunctional Aldh2*2. In other words, our data support the strategy that limiting alcohol drinking may reduce the ESCC risk in such individuals [10].

We have previously shown that alcohol drinking induces DNA adduct formation and DNA strand breaks in the esophageal squamous epithelium in *Aldh2*^*–/–*^ mice [53]. In cell culture, short-term acetaldehyde exposure leads to DNA adduct formation and DNA damage as well as various cellular responses such as apoptosis [53,54]. Our present data demonstrating that increased colony formation capability acquired during long-term acetaldehyde exposure may be accounted for by acetaldehyde-induced mutations of *TP53* and transforming genes that permit cells to negate acetaldehyde-induced genotoxic stress. Because mutagenic DNA adducts form following direct acetaldehyde exposure in the esophageal epithelium [55,56], chronic alcohol exposure is considered to lead to significant genomic instability in individuals with *Aldh2* dysfunction.

Furthermore, we have shown that alcohol drinking induced *TP53* mutations in the esophageal epithelium of *Aldh2*-deficient mice. As previously shown, *TP53* mutations occur not only in ESCC but also in the noncancerous esophageal epithelium due to aging and ingestion of risk factors [19]. Moreover, the frequency of *TP53* mutations is high in the epithelium with more Lugol-voiding lesions than those without these mutations [10]. We also previously reported that *TP53* mutations were essential factors for the transformation of esophageal epithelium [33,57]. Thus, *TP53*-gene mutations are considered to be an important genetic change in the early stage of esophageal carcinogenesis. Our data demonstrating that alcohol drinking alone in *Aldh2*-null mice did not induce ESCC supports the importance of *TP53* loss of function in the development of ESCC.

**Fig. 6:**
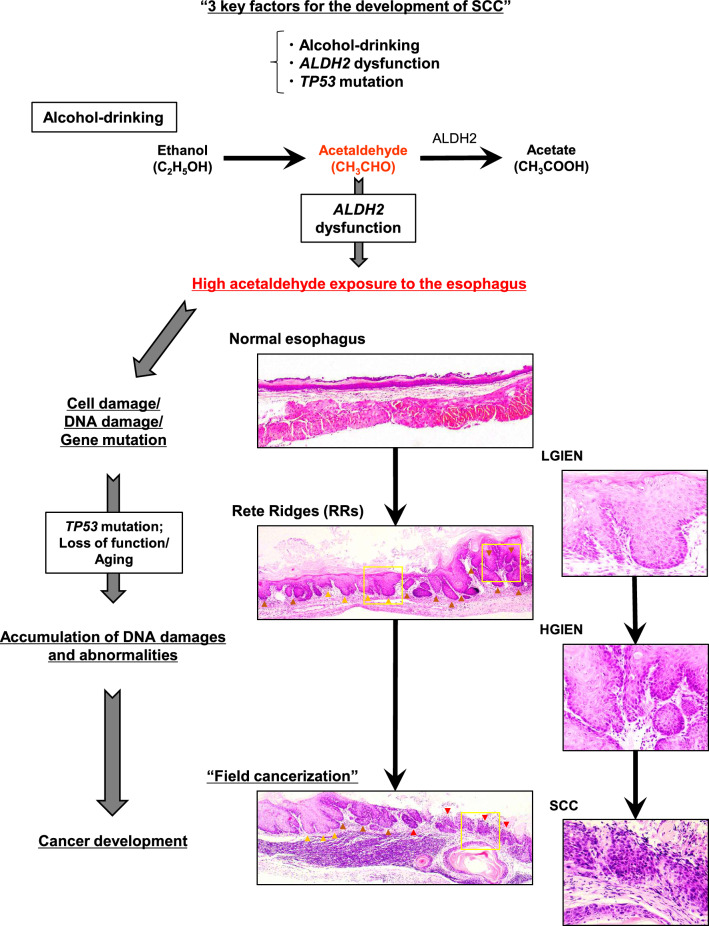
Image of esophageal field carcinogenesis caused by alcohol. Pathophysiology of alcohol-induced SCC development. When alcohol is consumed, EtOH is metabolized to acetaldehyde, and the acetaldehyde is detoxified to acetic acid by aldehyde dehydrogenase 2 (ALDH2). If the *ALDH2* gene polymorphism is impaired in this process, acetaldehyde is not degraded, resulting in exposure of the squamous epithelium to high concentrations of acetaldehyde. The mutagenicity of acetaldehyde causes cell damage, DNA damage, and gene mutations in the esophageal epithelium. The *Trp53* mutation, a typical gene mutation, causes characteristic morphologic changes (Rete ridges: red arrowheads are SCC, brown arrowheads are HGIEN, orange arrowheads are LGIEN) in the esophageal epithelium as the gene mutation accumulates with age, and changes such as LGIEN and HGIEN occur. These lesions are multiple and eventually lead to invasive carcinoma. The squamous epithelium by yellow rectangles in the center panel were enlarged in the right panels. This condition may reflect field carcinization, indicating that alcohol consumption, *Aldh2* deficiency, and *Trp53* mutation are essential key factors in squamous field cancerization

Furthermore, we showed that long-term acetaldehyde exposure to the esophageal epithelium resulted in an increased colony formation activity in vitro, suggesting that long-term acetaldehyde exposure generated squamous epithelial cells with enhanced anchorage-independent proliferative capability. Moreover, we revealed that long-term alcohol consumption induced various genetic abnormalities, including *TP53* gene, in the esophageal epithelium in *Aldh2*-deficient mice. These results indicate that long-term alcohol drinking in individuals with *Aldh2* dysfunction induces the accumulation of genetic abnormalities, which will increase the risk of squamous cancer development. The prevalence of G to A mutations is consistent with previous reports of gene mutation pattern in ESCC [18,58]. We summarize the data regarding our experimental model of alcohol-induced field cancerizations as follows. Chronic alcohol exposure in *KTPA*^*–/–*^ (*TP53*^*–/–*^; *Aldh2*^*–/–*^) mice induces squamous epithelial cell atypia associated with widespread RR formation to promote field cancerization (Fig. 6). Importantly, our data suggest that *Aldh2* and *Trp53* cooperate to develop alcohol-induced SCC development where Aldh2 mitigates acetaldehyde-mediated genomic toxicity whereas TP53 regulates DNA damage repair/cell-cycle checkpoint functions.

This study had several limitations. The first is that gene mutation analysis of mice esophagus after alcohol drinking has only resulted from target sequence, but this is due to the economic circumstances in our laboratory, and further extensive analysis will be necessary in the future. Second, our study cannot determine whether alcohol, *TP53* and *Aldh2* deficiency affect the cancer development of columnar epithelium in the esophagus, because the mice used in this study were cytokeratin *Krt5*-specific *TP53* deficient mice. At least, there were no cases in which tumors were found in the glandular (distal) -stomach. These results are consistent with clinical situations in which cancer development from squamous metaplasia of columnar epithelium is extremely rare in alcohol-induced SCC [45,46].

In conclusion, we for the first time clearly demonstrated that alcohol drinking together with loss of function of *Aldh2* and *Trp53* induced field carcinogenesis in the esophagus. This result might elucidate the mechanism of alcohol-induced squamous field cancerization as well as providing a basis for the development of cancer prevention.

## Supplementary Information

Below is the link to the electronic supplementary material.

Supplementary file1 (DOCX 2929 KB)

## Abbreviations

**AA**: Acetaldehyde
**Aldh2**: Aldehyde-dehdrogenase2
**ESCC**: Esophageal squamous cell carcinoma
**EtOH**: Ethanol
**HGIEN**: High-grade intraepithelial neoplasia
**H&E**: Hematoxylin and eosin
**IEN**: Intraepithelial neoplasia
**KSFM**: Keratinocyte serum-free media
**KTP**: Krt5Cre^ERT2^; Rosa26tdTomato^loxP^^−^^stop^^−^^loxP/loxP^^−^^stop^^−^^loxP^; Trp53^loxP/loxP^
**KTPA**: Krt5Cre^ERT2^; Rosa26tdTomato^loxP^^−^^stop^^−^^loxP/loxP^^−^^stop^^−^^loxP^; Trp53^loxP/loxP^; Aldh2
**LGIEN**: Low-grade intraepithelial neoplasia
**RRs**: Rete ridges
**SCC**: Squamous cell carcinoma
**TAM**: Tamoxifen
