# Supplementary material for: Aldh2 and the tumor suppressor Trp53 play important roles in alcohol-induced squamous field cancerization
Source: J Gastroenterol. 2025 Feb 6;60(5):546–60. doi: 10.1007/s00535-024-02210-y (PMC12014750; doi:10.1007/s00535-024-02210-y)
Supplement: Supplementary file 1 — Supplementary file1 (DOCX 2929 KB) [file 535_2024_2210_MOESM1_ESM.docx]

**Supplementary Figure**

**
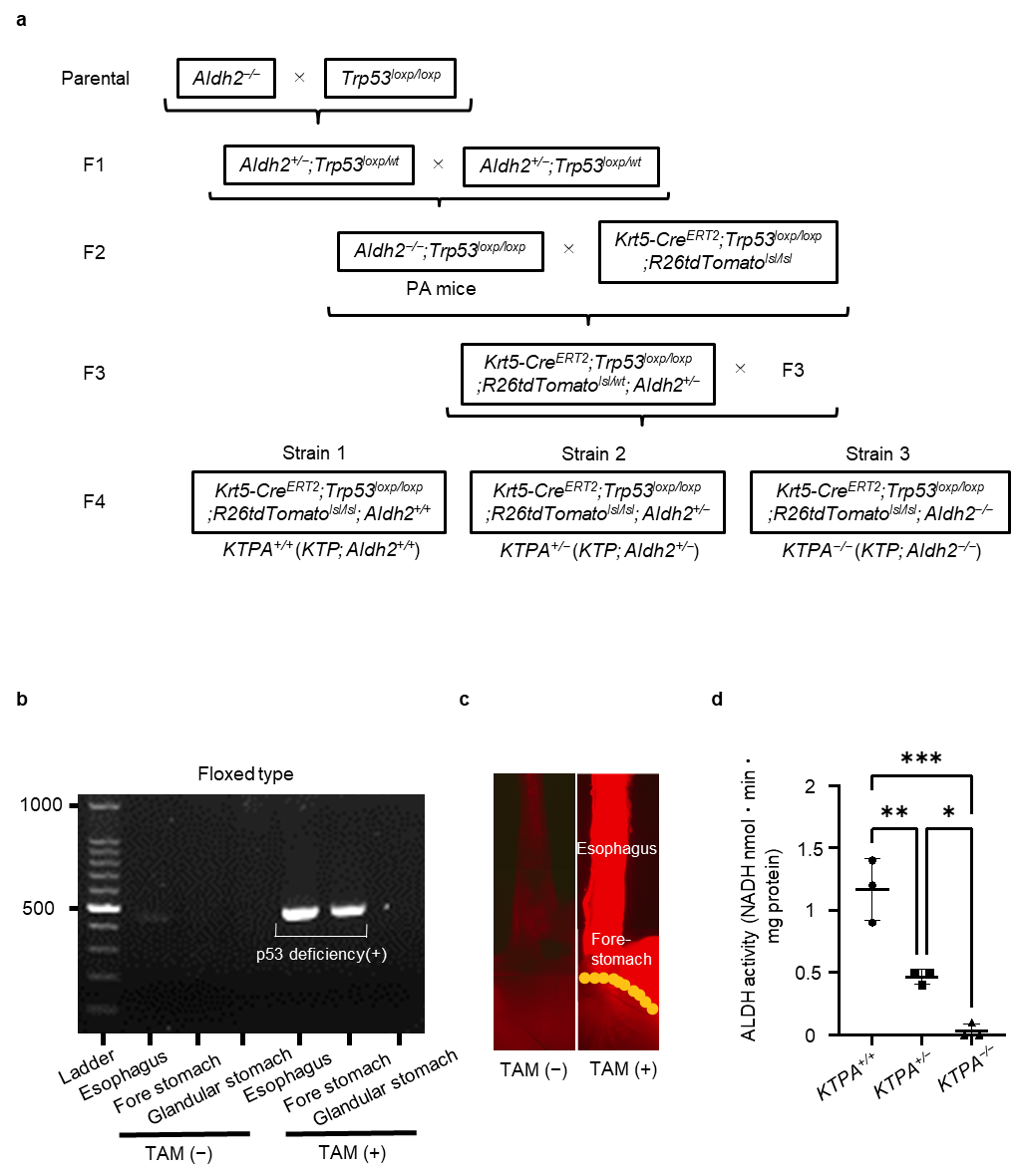
**

Supplementary Figure 1. (Breeding strategy and) Basic characterization of *KTPA* strains. (**a**) Mice with indicated genotype were crossed to produce *KTPA^+/+^*, *KTPA^+/–^*, and *KTPA^–/–^* strains used in this study. (**b**) Tamoxifen (TAM)-induced *K5Cre^ERT2^*-mediated *Trp53* deletion in *KTPA^–/–^* (*TP53^–/–^*; *Aldh2^–/–^*) mice. Genotyping was done by PCR (Fig. S5) on DNA purified from esophageal, forestomach, and glandular stomach epithelia from mice treated with or without TAM. The PCR product indicates Cre-mediated recombination of the conditional *Trp53^loxP/loxP^* alleles. **(c**) tdTomato expression in the esophagus and forestomach detected under fluorescence dissection microscopy in TAM-treated *KTPA^–/–^* (*TP53^–/–^*; *Aldh2^–/–^*) mice. The orange circles indicate the squamocolumnar junction (SCJ) between the forestomach and distal (glandular) stomach. **(d**) Hepatic Aldh activity in *KTPA^+/+^*, *KTPA^+/–^*, and *KTPA^–/–^*strains (*n* = 3 per strain). One-way ANOVA (P < 0.05), followed by post hoc Tukey’s multiple comparison test. Error bars represent mean ± SD. *P < 0.05, **P < 0.01, ***P < 0.001.

**
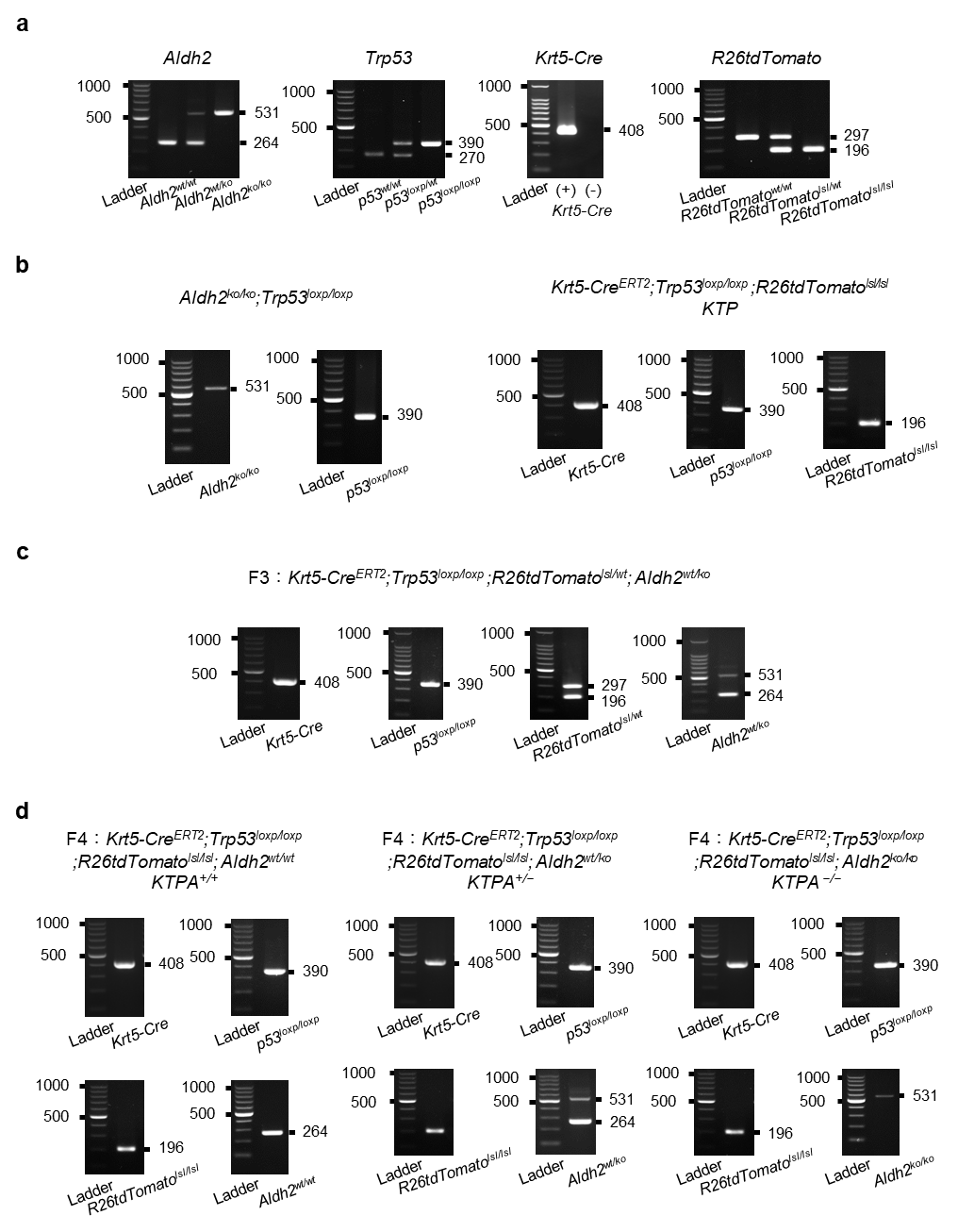
**

Supplementary Figure 2. PCR genotyping of genetically engineered mice. Genotyping was performed using PCR to document indicated genotypes in each mouse strain used in this study. PCR products were run on agarose gels along with a 100-base pair (bp) DNA ladder as a size marker and visualized by ethidium bromide staining for documentation. (**a**) Parental strains and derivatives carrying indicated genetically engineered alleles (*Aldh2^–/–^*, *Trp53^loxP/loxP^*, *Krt5Cre^ERT2^*, and *Rosa26tdTomato^lsl/lsl^*) in either homozygosity or heterozygosity, or a wild-type allele from the *C57BL/6* strain; (**b**), *Trp53^loxP/loxP^*; *Aldh2^–/–^* (TP), an intermediate and *KTP* strains; (**c**), *KTPA^+/–^* derived from intercrossing of *TP* and *KTP*; (**d**), *KTPA^+/+^*, *KTPA^+/–^*, and *KTPA^–/–^* strains generated by mutual intercrossing of *KTPA^+/–^* in (**c**). Expected size of PCR products is indicated for each gene.

**
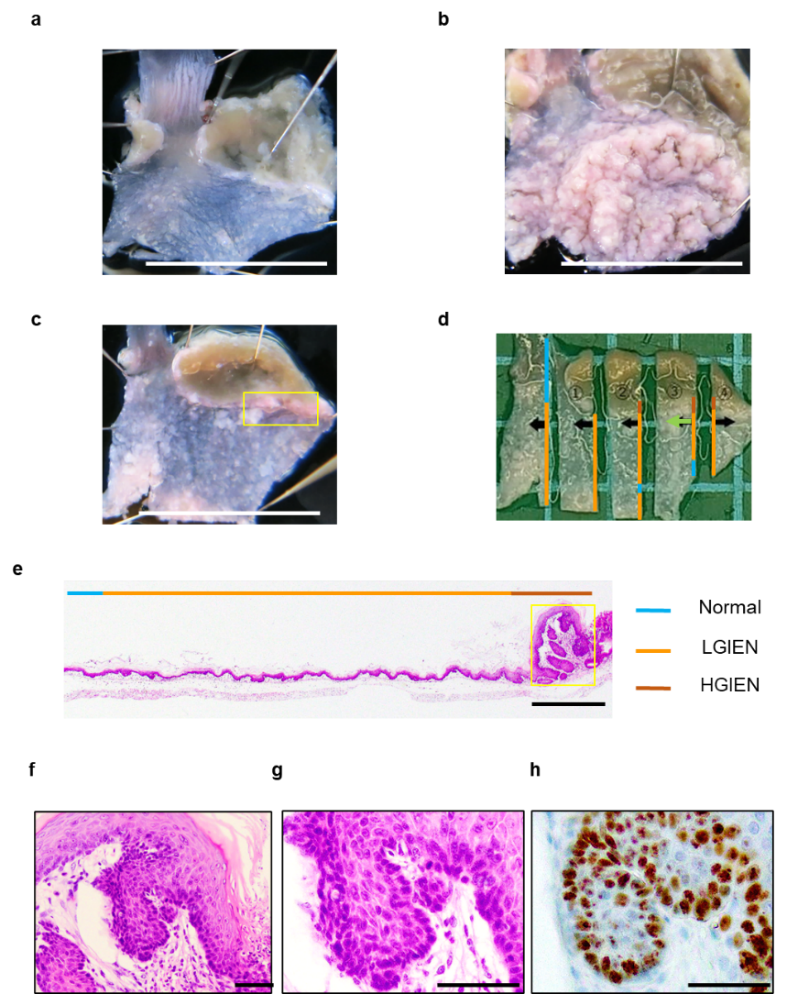
**

Supplementary Figure 3. Histopathological analyses of additional TAM-treated *KTPA*^–/–^ (*TP53^–/–^*; *Aldh2^–/–^*) mice with or without EtOH exposure. Representative macroscopic and microscopic images of dissected esophagus and forestomach samples collected from TAM-treated *KTPA^–/–^* (*TP53^–/–^*; *Aldh2^–/–^*) mice that received drinking water containing no EtOH for 34 weeks in (**a**), 10% EtOH for 34 weeks in (**b**) or 28 weeks in (**c, d, e, f, g and h**). Note that the mucosal surface from EtOH-treated mice displays protruded gross pinkish lesions at the SCJ (yellow rectangle) that may reflect an accumulation of tdTomato-positive cells and mucosal thickening that is not visible in the EtOH-untreated control animals. The specimen in (**c**) was grossed and serially sectioned in the direction indicated by black arrows in (**d**). Each section was subjected to histopathological mapping of normal, low-grade IEN (LGIEN), and high-grade IEN (HGIEN) lesions as indicated by a color code. The green arrow in (**d**) indicates the direction of cross sections utilized for the representative H&E-stained slide in (**e**). The HGIEN lesion at the SCJ (yellow rectangle) in (**c** and **d**) was enlarged in (**f** and **g**). The corresponding serial section was subjected to Immunohistochemistry (IHC) for Ki67 in (**h**). Scale bars = 1 mm in (**a, b and c**), 0.5 mm in (**d**), and 100 μm in (**e, f, g and** **h**).

**
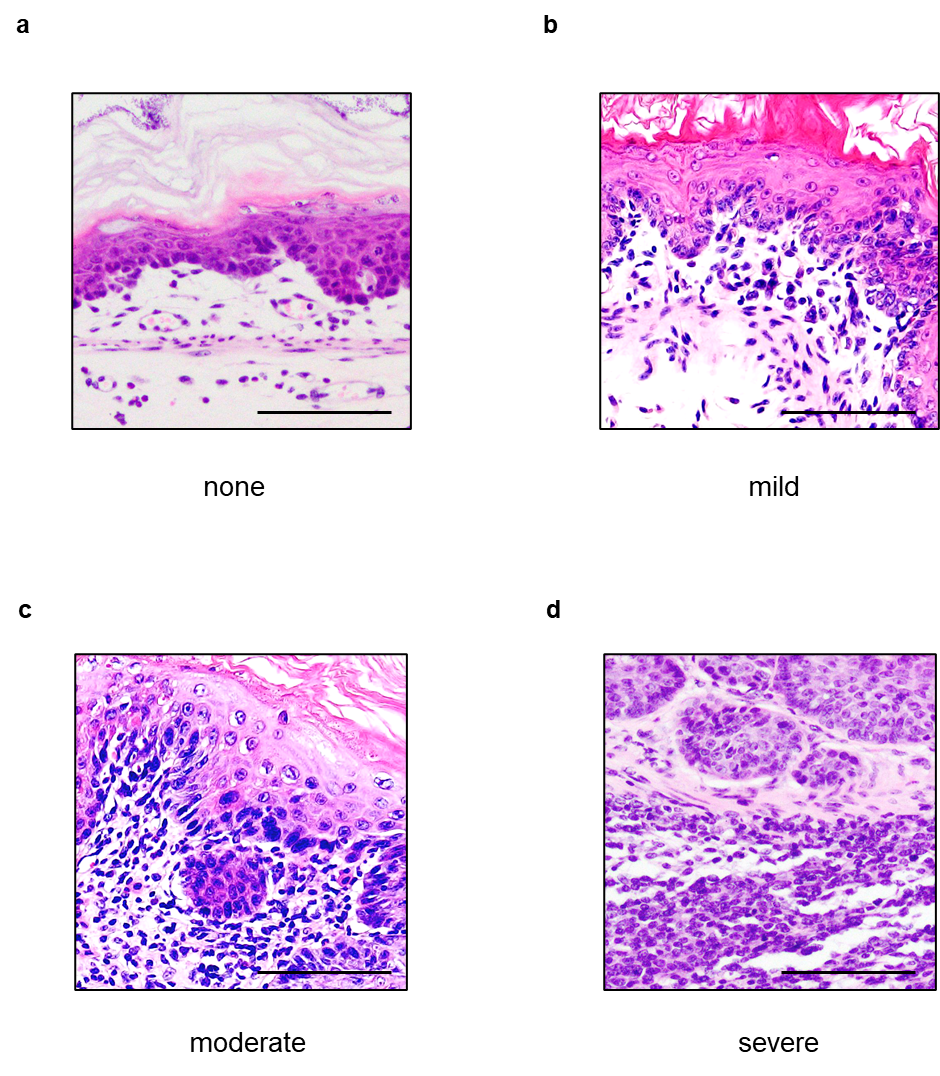
**

Supplementary Figure 4. Evaluation of inflammatory cell infiltration into the forestomach of each mouse group. Representative H&E images of the forestomach epithelium with inflammatory cell infiltration. Scale bar = 100 μm. A four-level score was given according to the degree of inflammation according to the frequency of inflammatory cells present in the visual field: (**a**) none (0 point); (**b**) <50%, mild (1 point); (**c**) 50%–80%, moderate (2 points); and (**d**) >80%, severe (3 points).

**Supplementary Table 2. Oligonucleotides used in the study.**

| Name | Sequence | Use | Notes |
| --- | --- | --- | --- |
| K5-Cre F | GCATTACCGGTCGATGCAACGAGTGATGAG | Genotyping | 408 bp for WT alleles |
| K5-Cre R | GAGTGAACGAACCTGGTCGAAATCAGTGCG | Genotyping |  |
| Trp53^flox^ F | GGTTAAACCCAGCTTGACCA | Genotyping | 390 bp for wt/wt alleles,  270 bp for loxp/loxp alleles |
| Trp53^flox^ R | GGAGGCAGAGACAGTTGGAG | Genotyping | 270 and 390 bp for loxp/wt alleles |
| mAldh2 F | GAGGACTGTGTTGGGAGGTC | Genotyping | 264 bp for wt/wt alleles |
| mAldh2 R1 | GTAGGTCCGGTCCCGTTC | Genotyping | 264 bp and 531 bp for wt/ko alleles |
| mAldh2 R2 | AGAAAGCGAAGGAACAAAGC | Genotyping | 531 bp for ko/ko alleles |
| R26tdTomato WT F | AAGGGAGCTGCAGTGGAGTA | Genotyping | 297 bp for wt/wt alleles |
| R26tdTomato WT R | CCGAAAATCTGTGGGAAGTC | Genotyping | 196 bp and 297 bp for lsl/wt alleles |
| R26tdTomato MT F | GGCATTAAAGCAGCGTATCC | Genotyping | 297 bp for lsl/lsl alleles |
| R26tdTomato MT R | CTGTTCCTGTACGGCATGG | Genotyping |  |
| Recombinate Trp53^flox^ F1 | CCAGCTTGACCATAGTGCCAT | Genotyping | around 500 bp for recombination of  the Trp53 gene |
| Recombinate Trp53^flox^ F2 | AAGGGGTATGAGGGACAAGG | Genotyping |  |
| Recombinate Trp53^flox^ R | TGGCTTCTACTATGGGTAGGG | Genotyping |  |
